# Changes in gravitational force affect gene expression in developing organ systems at different developmental times

**DOI:** 10.1186/1471-213X-5-10

**Published:** 2005-05-31

**Authors:** Naoko Shimada, Gbolabo Sokunbi, Stephen J Moorman

**Affiliations:** 1 Robert Wood Johnson Medical School, Department of Neuroscience and Cell Biology, 675 Hoes Lane, Piscataway, NJ 08854, USA

## Abstract

**Background:**

Little is known about the affect of microgravity on gene expression, particularly *in vivo *during embryonic development. Using transgenic zebrafish that express the *gfp *gene under the influence of a β-actin promoter, we examined the affect of simulated-microgravity on GFP expression in the heart, notochord, eye, somites, and rohon beard neurons. We exposed transgenic zebrafish to simulated-microgravity for different durations at a variety of developmental times in an attempt to determine periods of susceptibility for the different developing organ systems.

**Results:**

The developing heart had a period of maximum susceptibility between 32 and 56 hours after fertilization when there was an approximately 30% increase in gene expression. The notochord, eye, somites, and rohon beard neurons all showed periods of susceptibility occurring between 24 and 72 hours after fertilization. In addition, the notochord showed a second period of susceptibility between 8 and 32 hours after fertilization. Interestingly, all organs appeared to be recovering by 80 hours after fertilization despite continued exposure to simulated-microgravity.

**Conclusion:**

These results support the idea that exposure to microgravity can cause changes in gene expression in a variety of developing organ systems in live embryos and that there are periods of maximum susceptibility to the effects.

## Background

Research has shown that the space environment can alter gene expression, cell structure and function, organ systems and the behavior of organisms. While some of these changes may constitute adaptation to the space environment, we do not have a comprehensive understanding of the mechanisms driving these changes, nor are we wholly aware of the changes themselves. This is especially true with respect to development. The zebrafish is a powerful model for studying vertebrate development. We have previously demonstrated that zebrafish embryos can be sustained for at least 5 days in simulated-microgravity using a bioreactor that NASA designed for cells in culture [1,2]. Using this bioreactor, we have begun to examine the effect of simulated-microgravity on gene expression in specific developing organ systems *in vivo *using transgenic zebrafish that express the green fluorescent protein gene (*gfp*) under the influence of different promoters [3]. The use of *gfp *as a 'reporter-gene' has two significant advantages; gene expression can be monitored directly in a live animal, and changes in morphology that might be due to changes in expression of other genes can easily be detected.

We have been judicious in our choice of transgenic fish. β-actin is an ubiquitous cytoskeletal protein and β-actin gene expression is not thought to be affected by microgravity in cells in culture. However, the experiments that were done on cells in culture involved microarray analysis. It is not clear that changes in expression levels that we have documented in zebrafish embryos [3] and others have documented in cultured endothelial cells in simulated-microgravity [4] would have been detected using microarrays because the changes would not meet the 1–2 fold change threshold established for significance using microarrays. It is possible that there were changes in β-actin gene expression in the cultured cells of the same magnitude that we have seen in zebrafish embryos. Those changes would not have been deemed significant.

With the advent of bioinformatics, there has been a natural tendency to try to maximize the amount of data from individual experiments performed in Space using microarray technology. However, gene microarrays and northern blots can give misleading information, especially if they are done using materials from whole embryos, because changes in minor organs might be masked. For instance, a dramatic increase in β-actin gene expression exclusively in the hypochord, might not be detected because it would be averaged out by the lack of any significant change in any other structure. Using fluorescence imaging of whole embryos and a *gfp*-reporter gene approach, changes in gene expression should be detectable regardless of the size of the organ. In addition, data from microarrays and northern blots must be normalized against the expression level for genes that we know are not influenced by the experimental conditions to yield quantitative measures of expression changes. However, we cannot predict which genes are not affected by changes in gravitational force *in vivo*. Therefore, the choice of 'housekeeping gene' for normalization of microarray data could bias the results. For example, it is not unusual to use β-actin expression for normalization. Clearly this would not have been a good choice if the present experiments had been done using either microarrays or northern blots.

In our previous study heart and notochord development were examined at a single developmental time point [3]. Heart development was examined because previous studies had clearly demonstrated that the adult cardiovascular system is affected by changes in gravitational force [5-7]. However, the effects of microgravity on a developing heart are still poorly understood. The notochord, in most vertebrates, has adult derivatives but as a structure is present only during development. The notochord has been implicated in patterning the developing vertebral column [8], spinal cord [9], somites [10], and gut [11] among other structures. Despite its importance in patterning developing organ systems, the effects of microgravity on the notochord remain unknown. We demonstrated that simulated microgravity had effects on gene expression in the heart and notochord [3] but because of the limited extent of the study, we were not able to determine whether there were specific developmental periods when the heart or notochord were differentially susceptible to the effects.

We have now extended our previous work to include both a greater range of developmental time frames and an analysis of effects on other organ systems. Interestingly, of all the organs studied, the heart and notochord showed the most dramatic changes in gene expression.

## Results

### Whole embryo (Table 1 & Figure 1A)

Since body length is a better predictor of developmental stage than age [12], we measured body length in all embryos/larvae. There was no significant difference between the length of embryos/larvae in any of the experimental groups compared to their age matched controls (Data not shown). This suggests that none of the exposure durations used for this study caused any general developmental delay or acceleration.

**Table 1:** Table of percent changes in fluorescence intensity in different organs/cell types that resulted from exposure to simulated-microgravity for different developmental times.

**Whole Embryo**				**Heart**				**Notochord**			
**hpf**	**mean**	**SEM**	**p-value**	**hpf**	**mean**	**SEM**	**p-value**	**hpf**	**mean**	**SEM**	**p-value**

**8–24**	-1.24	2.25	0.37	**8–24**				**8–24**	0.58	4.51	0.25
**8–32**	8.40	3.61	0.07	**8–32**				**8–32**	16.07	5.60	0.03
**8–56**	11.59	3.66	0.003	**8–56**	9.41	8.12	0.17	**8–56**	10.11	6.23	0.08
**24–48**	8.81	2.86	0.01	**24–48**	12.19	5.83	0.04	**24–48**	16.17	4.56	0.003
**24–72**	18.62	4.58	0.0004	**24–72**	27.75	4.82	0.00003	**24–72**	31.68	6.80	0.0001
**24–80**	10.07	5.72	0.11	**24–80**	11.15	6.44	0.13	**24–80**	17.77	8.11	0.06
**32–56**	2.67	5.19	0.29	**32–56**	35.60	12.44	0.02	**32–56**	2.25	7.29	0.32
**32–80**	1.21	4.14	0.43	**32–80**	0.38	5.97	0.48	**32–80**	3.91	7.17	0.35
**48–72**	1.97	2.50	0.28	**48–72**	8.42	3.96	0.04	**48–72**	4.13	4.51	0.22
**56–80**	-2.58	2.82	0.27	**56–80**	5.01	5.61	0.27	**56–80**	-7.11	4.35	0.15
**Rohon Beard Neurons**				**Eye**				**Lens**			

**hpf**	**mean**	**SEM**	**p-value**	**hpf**	**mean**	**SEM**	**p-value**	**hpf**	**mean**	**SEM**	**p-value**

**8–24**				**8–24**	2.07	4.80	0.40	**8–24**			
**8–32**				**8–32**	3.01	5.23	0.36	**8–32**			
**8–56**	9.65	5.84	0.08	**8–56**	14.06	6.73	0.04	**8–56**	17.73	6.69	0.01
**24–48**	8.05	4.65	0.09	**24–48**	12.65	4.57	0.01	**24–48**	10.75	4.74	0.03
**24–72**	17.29	5.08	0.01	**24–72**	15.25	7.18	0.03	**24–72**	18.55	7.28	0.02
**24–80**	14.36	7.28	0.09	**24–80**	11.27	7.78	0.15	**24–80**	10.41	7.78	0.18
**32–56**	1.90	6.78	0.40	**32–56**	8.96	8.58	0.18	**32–56**	11.73	9.13	0.14
**32–80**	-1.60	5.72	0.41	**32–80**	0.70	5.94	0.47	**32–80**	-4.09	5.57	0.34
**48–72**	5.53	4.10	0.17	**48–72**	-3.77	3.73	0.24	**48–72**	-4.73	4.52	0.22
**56–80**	-4.76	4.30	0.24	**56–80**	1.25	5.06	0.44	**56–80**	2.83	5.91	0.37
**Somites**											

**hpf**	**mean**	**SEM**	**p-value**								

**8–24**	4.28	3.38	0.23								
**8–32**	4.10	5.71	0.31								
**8–56**	7.07	5.25	0.13								
**24–48**	7.50	4.52	0.09								
**24–72**	22.24	6.30	0.002								
**24–80**	15.39	7.80	0.08								
**32–56**	3.11	7.17	0.32								
**32–80**	2.08	5.89	0.41								
**48–72**	8.24	6.52	0.12								
**56–80**	-6.88	3.50	0.13								

hpf = hours post-fertilization that the embryos were kept in the bioreactor (see Figure 2);

SEM = standard error of the mean.

**Figure 1:**
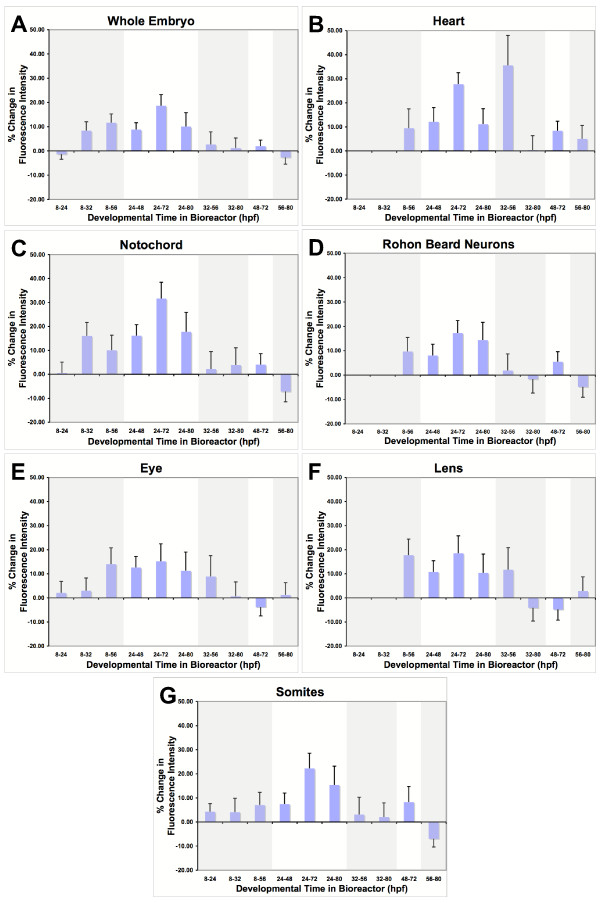
**Graphs of percent changes in fluorescence intensity in different organs/cell types that resulted from exposure to simulated-microgravity for different developmental times**. For each embryo, fluorescence intensity was measured in the regions illustrated in Figure 3. Each graph represents the changes in a specific organ/cell type. Regions of each graph are shaded to indicate exposure times that began at the same developmental time. All graphs are presented with the same exposure-time sequence on the x-axis and the same scale on the y-axis. Error bars = SEM

A brief exposure to simulated-microgravity during very early development (8–24 hpf) had no significant effect on overall fluorescence intensity of the whole embryo nor did any exposure that began after 32 hpf. However, exposure durations of at least 24 hours beginning prior to 32 hours caused increases in fluorescence with a peak occurring after exposure from 24 to 72 hours after fertilization. After 72 hours, continued exposure did not induce a further increase in expression but rather expression decreased. This suggests that a younger embryo is more susceptible to the effects of simulated-microgravity and that as the embryo matures, it develops mechanisms to adapt to simulated-microgravity.

### Heart (Table 1 & Figure 1B)

Gene expression in the heart appeared to follow the same general trend seen in the embryo as a whole except the changes were more dramatic. The heart showed changes that increased in magnitude with a peak after 72 hours followed by recovery despite continued exposure to simulated-microgravity. In addition, the developing heart had a period between 32 and 56 hours when it showed the most dramatic increase in fluorescence intensity, 35.60 +/- 12.44% (mean +/- SEM), while the embryo as a whole showed no change.

### Notochord (Table 1 & Figure 1C)

Gene expression in the notochord also appeared to follow the same general trend seen in the embryo, as a whole except, like the heart, the changes were more dramatic. In addition, the notochord, like the heart, showed a variation from the pattern of effects seen in the whole embryo. Unlike the heart, which showed a period of later susceptibility, the notochord had an additional period of earlier susceptibility (8–32 hpf) than the embryo as a whole.

### Rohon beard neurons, eye, lens, and somites (Table 1 & Figure 1D–G)

Gene expression in the rohon beard neurons, eye, lens, and somites followed the same general trend seen in the embryo as a whole.

## Discussion

The interpretation of the results presented here is based on the assumptions that *gfp *mRNA transcription, mRNA stability, and translation are similar to that of the native β-actin gene, and that an increase in GFP expression would be correlated with a similar increase in β-actin expression. This has been shown to be true for many other transgenic zebrafish (for instance see: [13-18]. Therefore, we can draw 3 general conclusions from the current work. First, simulated-microgravity can have global effects on gene-expression. Second, different organs/tissues, such as the heart and notochord, are more susceptible to the effects of simulated-microgravity on gene expression. Third, there are organ/tissue-specific periods of greater susceptibility that coincide with specific developmental events.

Simulated-microgravity's global effects on gene expression are suggested by the trends that become apparent when all the panels in Figure 1 are compared. For instance, early exposures of 24 to 48 hour durations tend to cause increases in β-actin gene expression in all the tissues examined. However, prolonging the exposure for durations longer than 48 hours and the embryos and tissues tend to show a recovery from the earlier effects. This is consistent with recent work using a similar bioreactor, showing that cultured endothelial cells up-regulate actin expression after early exposure to simulated-microgravity and recovery after prolonged exposure [4]. In addition, younger embryos tend to be more susceptible to the effects of simulated-microgravity on β-actin gene expression than older embryos (compare exposure times beginning at either 8 or 24 hours with exposure times beginning at 32 hours). In general, younger embryos are more susceptible and the effects are reversible.

Of all the organs/tissues that we have analyzed, the heart and notochord showed the most dramatic changes in β-actin gene expression after exposure to simulated-microgravity. Both of these organs showed periods of greater susceptibility, but the periods were different for the two organs. The heart, unlike the rohon beard neurons, the eye, the lens and the somites is involved in active, physical processes. This supports the idea that it is some aspect of the heart contraction/pumping that causes the more dramatic changes in the heart and could explain the smaller, similar changes in gene expression seen in the other tissues. The heart has a period of peak sensitivity between 32 and 56 hours post fertilization. This is the developmental time frame during which the beating heart tube is changing its shape as folding occurs and the atrio-ventrcular septum begins to form [19]. This might also be the developmental period during which the developing heart is most susceptible to the morphological influences of changes in shear forces associated with blood flow [20,21]. Since fluid dynamics are known to be influenced by changes in gravitational force, it is possible that the changes in β-actin gene expression could be due to changes in shear forces associated with blood flow through the heart in simulated-microgravity. Shear forces are also known to impact gene expression in cultured endothelial cells [21]. If simulated-microgravity related changes in shear force are initiating the change in gene expression in the zebrafish heart, then microgravity might have an impact on gene expression in the heart and blood vessels of astronauts during space flight. This raises the possibility that effects of microgravity on gene expression in endothelial cells *in vivo *might be a contributing factor in cardiovascular changes in astronauts.

The notochord shows more substantial early changes in β-actin gene expression than the developing heart and also appears to have two different periods of susceptibility (Figure 1C). We proposed above that the effects on the developing heart might be due to changes in shear forces associated with changes in fluid dynamics. The changes seen in the notochord are more difficult to explain. If intracellular micro-convective currents are significantly reduced in simulated-microgravity, actin polymerization-depolymerization could be affected because of a change in the local availability of actin subunits. In this case, the change in β-actin gene expression could reflect an attempt to maintain a stable cytoskeleton. However, the vector averaged nature of the bioreactor simulating microgravity might still allow for micro-convective currents that would play a role in regulating polymerization-depolymerization of actin filaments. If this is the case, then the notochord might be more susceptible to direct effects of microgravity than any other part of the embryo. The notochord is the major support structure of the embryo serving the same purpose as the vertebrae in older zebrafish. The two periods of susceptibility coincide with the 'straightening' of the embryo and the onset of spontaneous muscle contractions and movements of the embryo. These are the two developmental periods that the notochord cells would need to strengthen their cytoskeleton to provide additional stiffness, during the first period, and support for the embryo during the second period. Changes in gravitational force would result in a change in the stiffness and support that would need to be provided by the notochord. The same changes in support requirements result in changes in the skeletal system in astronauts during space flight suggesting that studying the zebrafish notochord in simulated-microgravity might provide insight into possible direct effects of microgravity on the skeletal system in astronauts.

## Conclusion

All-in-all, our results support the idea that exposure to microgravity can cause changes in gene expression in a variety of developing organ systems in live embryos and that there are periods of maximum susceptibility to the effects. In addition, the periods of susceptibility differ for different organ systems. Interestingly, organs that show effects in these experiments either develop into or regulate the development of organ systems that are affected in astronauts during space flight [22-24]. This suggests that ground-based research using zebrafish embryos might have predictive value for the affects of long-duration space flight on astronaut health.

## Methods

### Animals

Adult zebrafish (strain: Tg(actin:egfp)zp1) that expressed *gfp *gene under the influence of the promoter/enhancer of the zebrafish β-actin gene [3] were maintained at 28°C in communal tanks on a 14/10 hour light/dark cycle. Eggs were collected within 3 hours of when they were laid and fertilized. 8 hours post-fertilization (hpf) the eggs were transferred to room temperature (20°C) and phenyl-thiourea (0.003%: Sigma, St Louis MO) was added to the water to inhibit pigment production. The eggs were maintained at room temperature for the duration of the experiments. All experiments were performed under an approved IACUC protocol (#I02-020-02).

### Simulated-microgravity

**Figure 2:**
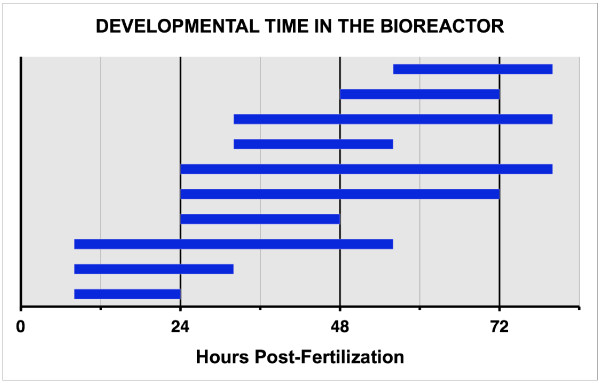
**Developmental time that embryos were in the bioreactor**. The bars indicate the developmental time, in hours post-fertilization, that embryos were kept in the bioreactor. N = 24 control & N = 24 Experimental for each exposure time.

To simulate many of the aspects of microgravity for zebrafish embryos and larvae, we used a bioreactor that NASA designed for cells in culture [1-3]. We placed the eggs in the bioreactor at the times indicated in Figure 2. 25–30 eggs were placed in the bioreactor at the beginning of each experiment. When eggs were removed from the bioreactor, 9 embryos were chosen at random and manually removed from the chorion. 9 embryos were prepared for imaging in case some of the embryos did not express the transgene. Only 6 embryos/larvae were used for acquiring images of GFP fluorescence. Each experiment was repeated 4 times to give a total of N = 24 in each group.

Before beginning the experiments we compared gene expression in embryos incubated in an identical bioreactor oriented in an upright position so that it functioned as a slow speed centrifuge with embryos incubated in a Petri dish placed on the support base of the bioreactor. The Petri dish with the embryos (N = 24) was placed on the support base of the bioreactor so that the embryos were exposed to the same temperature, light cycle, and vibration as the embryos in the bioreactor. Embryos (N = 24) were placed in the bioreactor at 24 hpf and maintained in the bioreactor for 48 hrs. There were no differences in morphology, or gene expression for the whole embryo (mean = -1.51; sem = 5.79; p = 0.43), the heart (mean = 1.13; sem = 6.28; p = 0.45), the notochord (mean = -1.49; sem = 6.21; p = 0.49), the eye (mean = -1.21; sem = 5.37; p = 0.49), the lens (mean = 0.41; sem = 6.57; p = 0.45), the somites (mean = 1.49; sem = 6.21; p = 0.49), or the rohon beard neurons (mean = 1.26; sem = 6.34; p = 0.47) between the two groups of embryos. Therefore, all subsequent experiments were performed using control embryos in a Petri dish on the support base of the bioreactor.

### GFP-fluorescence imaging

All images were collected using a Leica DMRE microscope (Leica Microsystems Inc., Bannockburn, IL) equipped with a Ludl BioPrecision motorized stage (Ludl Electronic Products Ltd., Hawthorne, NY) and a Hamamatsu Orca-ER camera (Hamamatsu Photonics, Hamamatsu City Japan). The microscope, stage, and camera were controlled using OpenLab software (Improvision, Lexington, MA) running on an Apple Dual-processor G4 computer. Prior to collecting fluorescence images, a bright-field image of each embryo/larvae was acquired using a 5× objective. This image was used to measure rostral-caudal length as an indication of the age of the embryo/larva. After the brightfield image was acquired, a complete Z-series of fluorescence images was acquired. For this series, images were collected at 3 μm intervals using a 10× objective. The entire stack of images was saved to disk. The camera gain, offset, and exposure time were kept constant for all homozygous embryos/larvae. Because heterozygous embryos/larvae had approximately half the fluorescence intensity as homozygous embryos/larvae, a second set of camera settings was used for all of the heterozygous embryos/larvae. Embryos older than 24 hpf were anesthetized using tricaine (0.04% 3-amino benzoic acidethylester: Sigma, St. Louis) during imaging to prevent movements of the embryo.

### GFP intensity measurements

**Figure 3:**
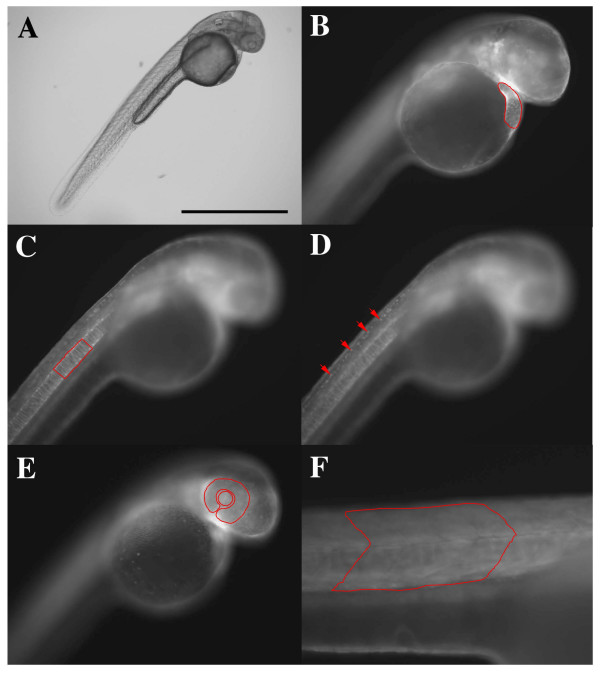
**Images of the same transgenic zebrafish embryo (strain: Tg(actin:egfp)zp1) 48 hours post fertilization**. **A. **Bright field image of the embryo. **B-D. **Fluorescence images of the same embryo at different focal planes. **B. **Image at the focal plane for heart measurement. The heart is outlined in red. **C. **Image at the focal plane for notochord measurement. A length of notochord adjacent to 4 somites is outlined in red. **D. **Image at the focal plane for rohon beard neuron measurement. 4 rohon beard neurons are indicated by red arrows. **E. **Image at the focal plane for eye and lens measurement. The eye and lens are outlined, separately, in red. **F. **Image at the focal plane for somite measurement. 4 somites are outlined in red. Scale bar = 1 mm (**A**), 0.5 mm (**B – E**), 0.05 mm (**F**).

Using the OpenLab software, an image where the organ of interest was in focus was selected from the z-series stack. A region of interest was then drawn around the organ (Figure 3) and the software automatically calculated the average intensity within the region. To estimate the average intensity of fluorescence for the entire embryo/larva, an image was selected from the middle of the z-series stack and the average intensity for the entire image was calculated.

### Data analysis and statistics

The measurements of fluorescence intensity in homozygous and heterozygous control embryos/larvae were used to normalize the measurements in homozygous and heterozygous experimental embryos/larvae respectively. After the individual measurements were normalized, the mean, standard deviation, and standard error of the mean were calculated for each group. The means for the control and experimental groups were compared using a t-test.

## Authors' contributions

NS carried out the majority of the experiments and participated in the interpretation of the results and drafting of the manuscript. GS carried out some of the experiments and participated in the design of the study. SJM conceived of the study, and participated in its design and coordination and helped to draft the manuscript. All authors read and approved the final manuscript.
